# Molecularly Engineered Nitrate Double Perovskites with Elevated Ferroelectric Phase Transition Temperature and Large Piezoelectric Response

**DOI:** 10.1002/advs.78404

**Published:** 2026-10-26

**Authors:** Le Ye, Guang‐Yuan Hu, Yarou Duan, Yutong Lin, Ding‐Chong Han, Zi‐Luo Fang, Zhe Dong, Wei‐Xiong Zhang, Liang‐Han Shen, Na Wang, Le‐Ping Miao, Heng‐Yun Ye, Lingling Mao

**Affiliations:** ^1^ Department of Chemistry Southern University of Science and Technology Shenzhen China; ^2^ SUSTech Energy Institute For Carbon Neutrality Southern University of Science and Technology Shenzhen China; ^3^ Chaotic Matter Science Research Center Jiangxi Province Key Laboratory of Functional Crystalline Materials Chemistry International Institute for Innovation Jiangxi University of Science and Technology Ganzhou China; ^4^ MOE Key Laboratory of Bioinorganic and Synthetic Chemistry IGCME School of Chemistry Sun Yat‐sen University Guangzhou China

**Keywords:** ferroelectric phase transitions, luminescence, molecular double perovskites, molecular engineering, piezoelectricity

## Abstract

Conventional *ABX*_3_ perovskites place organic species at *A*‐sites or as *X*‐site linkers, where the *B*‐site is reserved for metals. Molecular double perovskites with a general formula *A*_2_ *B* ^I^ *B* ^III^ *X*_6_ possess higher tunability and distinct *B* ^I^‐sites that can accommodate small cations, further regulating lattice connectivity and physical properties. Here, we introduce a new family of *B* ^I^‐site‐organic nitrate double perovskites, (RM3HQ)_2_ *B* ^I^Eu(NO_3_)_6_ (RM3HQ = (*R*)‐*N*‐methyl‐3‐hydroxyquinuclidinium), forming compounds **1** **–** **4** with *B* ^I^ = ammonium, methylammonium, formamidinium, and dimethylammonium, respectively. Compounds **1** and **2** adopt mixed corner‐/face‐sharing non‐perovskite structures, whereas **3** and **4** form double perovskites. This structural variation raises the ferroelectric phase‐transition temperature to 349 K in **4** and 414 K in **3**, much higher than that of the inorganic analogue (*T*_c_ = 285 K). This further widens the temperature margin for stable room‐temperature piezoelectric responses, as demonstrated in **3** by ferroelectric hysteresis loops, ferroelastic domains, and a large piezoelectric coefficient (|*d*_33_| ≈ 56 pC N^−1^). During these phase transitions, the Eu^3+^ photoluminescence intensity ratio (616 nm/ 592 nm) increases from 0.32 to 2.92 in **3** and from 0.37 to 4.59 in **4**, reflecting transition‐related changes in the ligand field. This work highlights the *B* ^I^‐site as an effective handle for tuning ferroelectric phase‐transition temperatures and tunable Eu^3+^ photoluminescence.

## Graphical abstract

This study reports molecularly engineered nitrate double perovskites based on systematic *B* ^I^‐site cation substitution. This molecular engineering modulates local *B* ^I^···NO_3_ interactions, nitrate connectivity, and collective physical responses, accompanied by structural conversion from mixed corner‐/face‐sharing structures to three‐dimensional corner‐sharing double perovskites. The resulting materials exhibit elevated ferroelectric phase‐transition temperatures, a large piezoelectric response, ferroelasticity, and Eu^3+^ luminescence responses, establishing *B* ^I^‐site molecular engineering as a strategy for multifunctional molecular materials.

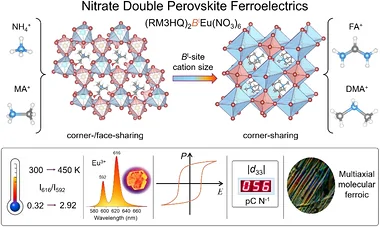

## 1 Introduction

Hybrid perovskites display rich structural diversity, in which subtle structural changes can produce pronounced physical responses [1, 2]. Molecular motion and octahedral connectivity are key factors that drive symmetry breaking, giving rise to ferroelectricity, piezoelectricity, ferroelasticity, and nonlinear‐optical responses [1, 2, 3, 4, 5, 6, 7, 8, 9]. In hybrid *ABX*_3_ perovskites, organic species can occupy the *A*‐site or serve as *X*‐site linkers in metal–organic variants [10, 11, 12, 13, 14, 15]. The *B*‐site, however, remains occupied by metal cations in *BX*_6_ octahedra.

Metal‐free perovskites have shown that ammonium cations can occupy the *B*‐site, but these materials do not contain metal‐centered *BX*_6_ octahedra [16, 17, 18, 19]. In this context, molecular double perovskites (*A*_2_ *B* ^I^ *B* ^III^ *X*_6_) can accommodate an organic ammonium cation at the *B* ^I^‐site and a metal cation at the *B* ^III^‐site. Reported examples are mainly one‐dimensional hexagonal double perovskites, which have shown tunable ferroelectric, antiferroelectric, and luminescent responses [20, 21, 22, 23]. However, three‐dimensional (3D) corner‐sharing structures with *B* ^I^‐site organic ammonium cations remain rare.

Nitrate‐based rare‐earth molecular double perovskites are well suited to this context because their *A*_2_ *B* ^I^[Ln(NO_3_)_6_] structures place Ln^3+^ at the *B* ^III^‐site and retain a distinct *B* ^I^‐site for small cations within a 3D corner‐sharing arrangement [24, 25, 26, 27, 28, 29, 30, 31, 32, 33, 34, 35, 36, 37, 38, 39, 40, 41, 42, 43, 44, 45]. These *B* ^I^‐site variations offer a means of tuning both the phase‐transition behavior and luminescence of Eu‐based nitrate molecular double perovskites. A representative example is a previously reported molecular double perovskite that exhibits ferroelectric and piezoelectric responses together with Eu^3+^ emission but undergoes a phase transition at only 285 K, leaving a narrow operating window for room‐temperature piezoelectric response [28]. This limitation motivates efforts to raise the transition temperature well above room temperature. Replacing the inorganic Rb^+^ at the *B* ^I^‐site with NH_4_^+^ or small organic ammonium cations introduces hydrogen bonding and thermally activated reorientational motion, which can in turn modulate the local *B* ^I^···NO_3_ interactions, nitrate connectivity, and ultimately the phase‐transition temperature [24, 25]. Such local structural changes can be probed by temperature‐dependent Eu^3+^ luminescence, where the PL intensity ratio between emissions at 616 and 592 nm (I_616_/I_592_) serves as an intrinsic optical signature of ligand‐field variations across the structural phase transitions [46, 47, 48].

In this work, guided by the chemical design principles of ferroelectrochemistry [49, 50], we chose the chiral (*R*)‐*N*‐methyl‐3‐hydroxyquinuclidinium (RM3HQ^+^) cation as the *A*‐site component, thereby establishing a common chiral molecular environment across the series. We prepared compounds **1–4** by varying the *B* ^I^‐site cation in the molecular double‐perovskite (RM3HQ)_2_ *B* ^I^Eu(NO_3_)_6_, using NH_4_^+^, methylammonium (MA^+^), formamidinium (FA^+^), and dimethylammonium (DMA^+^), respectively. These *B* ^I^‐site cations differ in molecular geometry, size, steric effects, and N–H donor arrangement, enabling us to examine how *B* ^I^‐site substitution influences nitrate connectivity and ferroic phase transitions. Compounds **1** and **2** adopt mixed corner‐/face‐sharing non‐perovskite structures, whereas **3** and **4** form 3D corner‐sharing double perovskites. The ferroelectric transition temperatures are elevated to 414 K in **3** and 349 K in **4**. Across these transitions, the Eu^3+^ emission ratio I_616_/I_592_ reflects the phase‐transition‐induced ligand‐field changes. These results establish the underexplored molecular tunability of the *B* ^I^‐site as an effective handle for tuning ferroic phase‐transition temperatures and for probing ligand‐field changes through Eu^3+^ luminescence in hybrid systems.

## 2 Results and Discussion

### 2.1 Synthesis and Crystal Structures

The four new compounds, (RM3HQ)_2_ *B* ^I^Eu(NO_3_)_6_, were obtained as colorless block‐shaped crystals, with *B* ^I^ = NH_4_^+^, MA^+^, FA^+^, and DMA^+^ for **1–4**, respectively (Figure S1; synthetic details in the Supporting Information). Experimental powder X‐ray diffraction patterns agree well with those simulated from the single‐crystal structures, confirming the phase purity of the bulk samples (Figure S2). Single‐crystal X‐ray diffraction (SCXRD) established the structures of **1–4**; crystallographic data and refinement parameters are compiled in Tables S1–S3.

The general formula can be described in terms of three structural components (Figure 1a): the chiral *A*‐site cation RM3HQ^+^, the Eu(NO_3_)_6_ coordination unit, and the variable *B* ^I^‐site cation. From the viewpoint of overall octahedral connectivity, SCXRD reveals two *B* ^I^‐dependent structure types (Figure 1b,c). Compounds **1** and **2** adopt Type I 3D non‐perovskite structures, featuring mixed corner‐ and face‐sharing connections between Eu(NO_3_)_6_ and *B* ^I^(NO_3_)_6_ units. In contrast, compounds **3** and **4** form Type II 3D corner‐sharing double‐perovskite structures. This difference is most clearly seen in the local *B* ^I^(NO_3_)_6_ units (Figure 1d). In **1** and **2**, the smaller NH_4_^+^ and MA^+^ cations organize three neighboring nitrate groups into a hydrogen‐bonded triad through directional N–H···O contacts, giving rise to the face‐sharing component. In **3** and **4**, the larger FA^+^ and DMA^+^ cations are associated with a more open nitrate arrangement and purely corner‐sharing connectivity. These observations suggest that the size and hydrogen‐bonding behavior of the *B* ^I^‐site ammonium cations control the local nitrate arrangement and overall connectivity.

**FIGURE 1:**
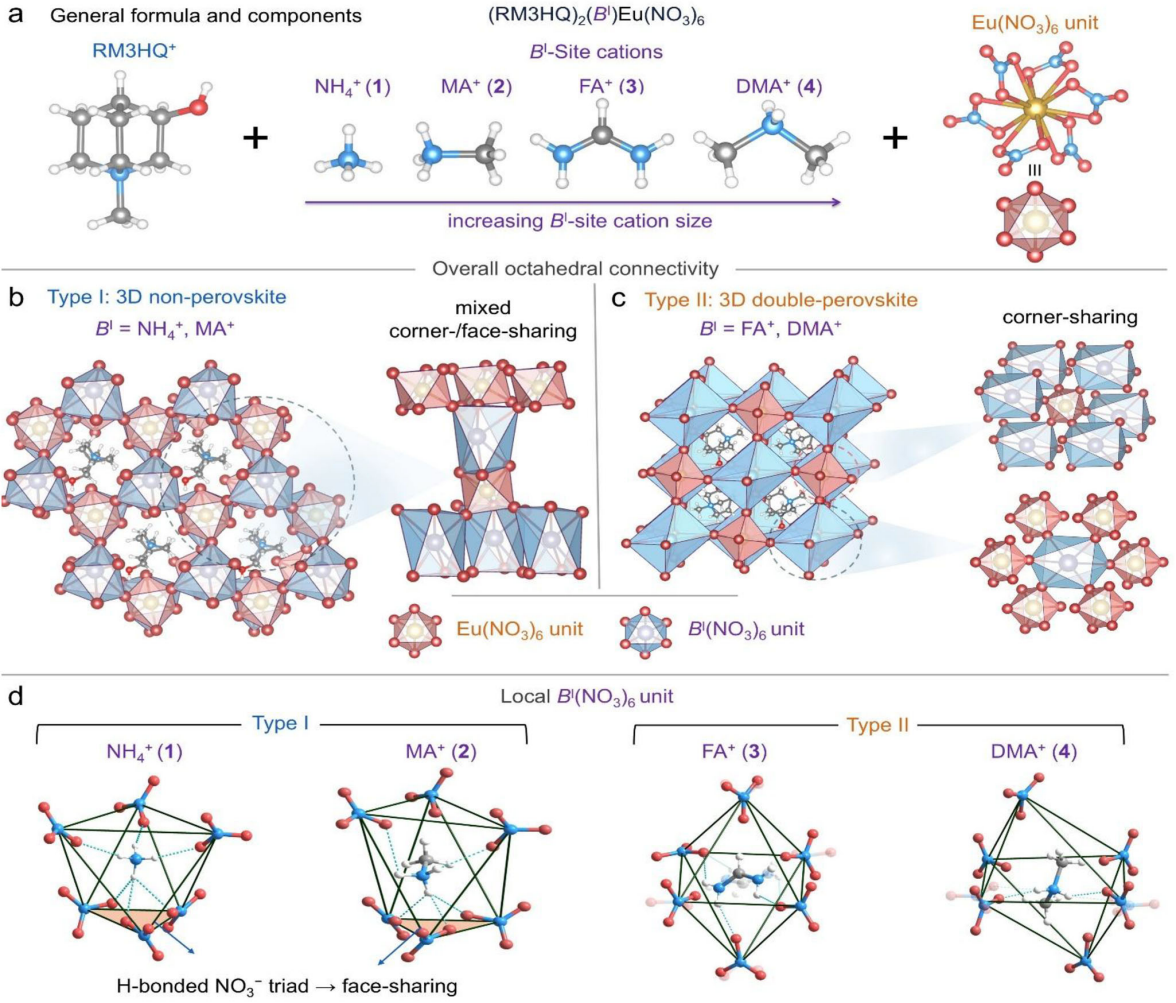
(a) General formula and structural components: the (*R*)‐*N*‐methyl‐3‐hydroxyquinuclidinium cation (RM3HQ^+^), *B* ^I^‐site cations, and Eu(NO_3_)_6_ units. (b,c) *B* ^I^‐dependent structure types: Type I 3D non‐perovskite structures for **1** and **2**, and Type II 3D double‐perovskite structures for **3** and **4**. (d) Local *B* ^I^(NO_3_)_6_ unit with N–H···O contacts shown as cyan dashed lines. In **1** and **2**, the orange shared face is formed by a hydrogen‐bonded NO_3_^−^ triad. Polyhedra: *B* ^I^(NO_3_)_6_, light blue; Eu(NO_3_)_6_, light orange. Atoms: C, gray; H, white; N, blue; O, red; Eu, yellow.

Compared with our previously reported Rb^+^ and K^+^ analogues, the overall connectivity correlates with the average *B* ^I^···Eu separation: shorter separations generally give mixed corner‐/face‐sharing connectivity, whereas longer separations correspond to purely corner‐sharing structures (Table S4) [28, 37]. However, the size of the *B* ^I^‐site cation is not the only factor governing connectivity selection. The La analogue, (RM3HQ)_2_NH_4_La(NO_3_)_6_, adopts a corner‐sharing double‐perovskite structure despite containing the same NH_4_^+^ cation as **1**, indicating that the lanthanide ionic radius also influences the resulting connectivity [25]. Thus, the present Eu‐based series highlights the role of *B* ^I^‐site substitution in regulating framework connectivity, while the influence of the Ln^3+^ ionic radius should also be considered when extending this strategy to related rare‐earth systems. Compounds **3** and **4**, with their well‐defined 3D corner‐sharing double‐perovskite structures, enable examination of the order–disorder dynamics discussed below.

### 2.2 Structural Phase Transitions of **3** and **4**

Variable‐temperature SCXRD reveals stepwise symmetry evolution in both **3** and **4**. Compound **3** adopts a polar *C*2 room‐temperature phase (RTP); on heating, it transforms through a nonpolar *R*32 intermediate‐temperature phase (ITP) into a cubic *F*432 high‐temperature phase (HTP), with the three phases characterized by SCXRD at 293, 420, and 433 K, respectively (Figure 2). Compound **4** undergoes the same *C*2 → *R*32 → *F*432 pathway, with its three phases characterized at 300, 353, and 373 K (Figure S5).

**FIGURE 2:**
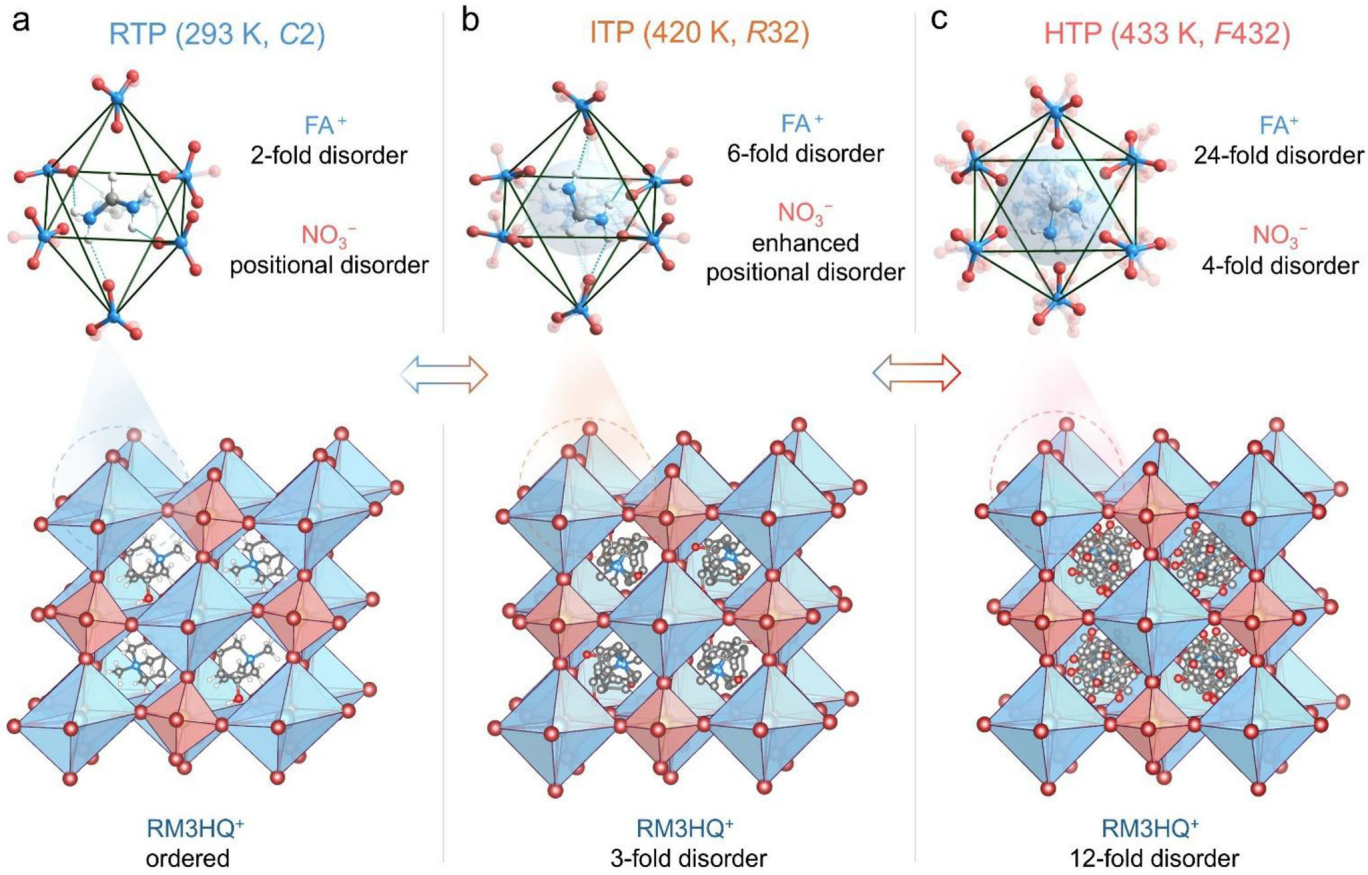
Variable‐temperature crystal structures of compound **3**. RTP, ITP, and HTP denote the room‐temperature, intermediate‐temperature, and high‐temperature phases, respectively. (a–c) Crystal structures of the RTP (293 K, *C*2), ITP (420 K, *R*32), and HTP (433 K, *F*432) phases, with local *B* ^I^(NO_3_)_6_ coordination environments and packing views shown for each phase. Red spheres denote nitrate groups; blue and pink octahedra denote *B* ^I^(NO_3_)_6_ and Eu(NO_3_)_6_ units; cyan dashed lines indicate N–H···O hydrogen bonds. Atom colors: C, gray; H, white; N, blue; O, red.

In compound **3**, the 3D corner‐sharing double‐perovskite structure is preserved throughout, and the transitions arise from the cooperative orientational and positional disordering of FA^+^, NO_3_^−^, and RM3HQ^+^ (Figure 2a–c). In the RTP (Figure 2a), FA^+^ is distributed over two orientations related by the crystallographic 2‐fold axis, four of the six NO_3_^−^ groups exhibit split‐site positional disorder, and RM3HQ^+^ remains fully ordered. Despite this local disorder, the polar space group *C*2 is maintained. Upon heating to the ITP (Figure 2b), FA^+^ accesses six orientations generated by the 3‐fold and 2‐fold axes, the NO_3_^−^ split‐site occupancies converge toward 0.5/0.5, and RM3HQ^+^ develops 3‐fold disorder around the 3‐fold axis. This concerted disordering elevates the average symmetry to *R*32 and eliminates the polar order. In the HTP (Figure 2c), FA^+^ occupies 24 symmetry‐related orientations generated by the 4‐fold, 3‐fold, and 2‐fold axes; NO_3_^−^ adopts a highly averaged distribution, and RM3HQ^+^ exhibits 12‐fold disorder. Across the full sequence, FA^+^ orientational disordering appears to dominate the local disorder, with NO_3_^−^ and RM3HQ^+^ responding cooperatively. This correlation suggests that *B* ^I^‐site motion is linked to nitrate rearrangement and contributes to the long‐range symmetry evolution.

Compound **4** follows the same pathway but starts from a more ordered configuration. In its RTP, DMA^+^ is fully ordered within the cavity, only two of the six NO_3_^−^ groups show split‐site positional disorder, and RM3HQ^+^ remains ordered as in the RTP of **3** (Figure S5). The ITP and HTP show disorder distributions analogous to those of **3**. Nevertheless, **4** undergoes the order–disorder transition at a substantially lower temperature than **3**. This difference can be attributed to the different local *B* ^I^(NO_3_)_6_ environments. FA^+^ is a rigid, planar cation that anchors to the surrounding nitrate groups through four directional N–H···O contacts and thus requires a relatively high temperature to activate its orientational disorder. DMA^+^, by contrast, is more flexible and nonplanar, with only two N–H donors flanked by methyl groups; its weaker and less directional *B* ^I^···NO_3_ interactions allow orientational disorder to set in at a lower temperature. Thus, the higher transition temperature of **3** relative to **4** reflects the stronger and more directional local *B* ^I^···NO_3_ interactions in the FA^+^‐centered *B* ^I^(NO_3_)_6_ unit.

### 2.3 Phase Transitions and Macroscopic Ferroic Responses

Differential scanning calorimetry (DSC) reveals that the two corner‐sharing double‐perovskite compounds, **3** and **4**, both show two reversible thermal anomalies with pronounced hysteresis (Figure 3a,b). For the FA^+^ compound **3**, two endothermic peaks appear at 414 and 420 K upon heating, with the corresponding exothermic peaks at 412 and 402 K upon cooling. The DMA^+^ analogue **4** displays a similar two‐step thermal profile but at substantially lower temperatures, with endotherms at 349 and 353 K and exotherms at 345 and 331 K.

**FIGURE 3:**
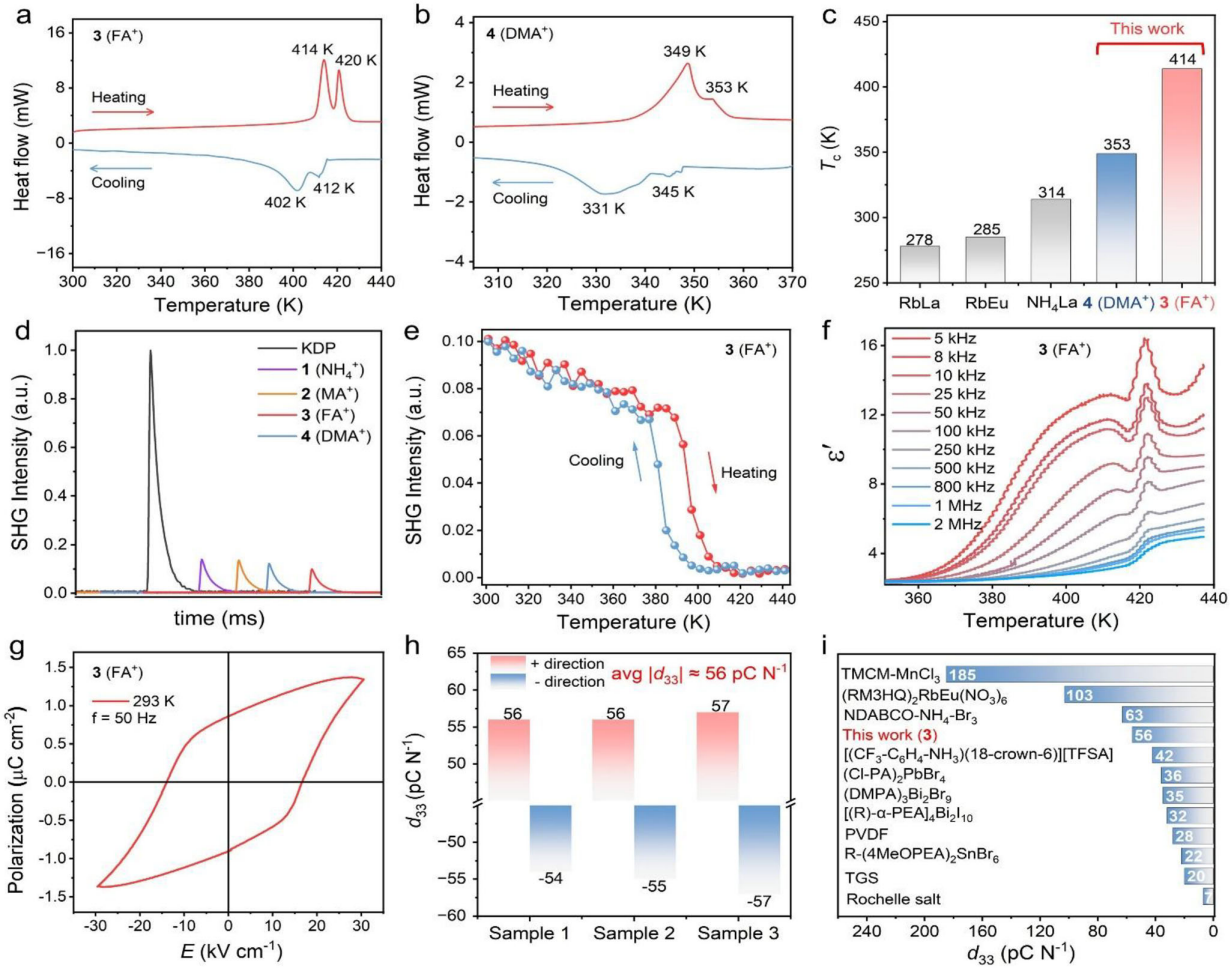
Phase transitions and macroscopic ferroic responses of (RM3HQ)_2_ *B* ^I^Eu(NO_3_)_6_. DSC curves of compounds **3** (a) and **4** (b) upon heating (red) and cooling (blue). (c) Comparison of the ferroelectric Curie temperatures (*T*_c_) of **3** and **4** with three isostructural analogues: RbLa = (RM3HQ)_2_RbLa(NO_3_)_6_ [25], RbEu = (RM3HQ)_2_RbEu(NO_3_)_6_ [28], and NH_4_La = (RM3HQ)_2_(NH_4_)La(NO_3_)_6_ [25]. (d) Powder SHG signals of compounds **1–4** at room temperature, with KDP as a reference; traces are horizontally offset for clarity. (e) Variable‐temperature SHG intensity of **3** upon heating (red) and cooling (blue). (f) Temperature‐dependent dielectric response (*ε*′) of **3** at frequencies from 5 kHz to 2 MHz. (g) Polarization–electric field (*P*–*E*) hysteresis loop of **3** at 293 K and 50 Hz. (h) Quasi‐static *d*_33_ values of **3** measured on three independent samples. (i) Comparison of *d*_33_ values of **3** with representative piezoelectric materials [3, 28, 51, 52, 53, 54, 55, 56, 57, 58, 59].

Within the corner‐sharing Eu series, replacing the inorganic Rb^+^ at the *B* ^I^‐site with the molecular cations DMA^+^ and FA^+^ raises the first polar‐to‐nonpolar transition temperature from 285 K in the previously reported (RM3HQ)_2_RbEu(NO_3_)_6_ analogue to 349 K in **4** and 414 K in **3**, corresponding to increases of 64 and 129 K, respectively [28]. This comparison demonstrates that molecular substitution at a single crystallographic *B* ^I^‐site can substantially elevate the polar‐to‐nonpolar transition temperature (Figure 3c). Two La‐based analogues, (RM3HQ)_2_RbLa(NO_3_)_6_ and (RM3HQ)_2_(NH_4_)La(NO_3_)_6_, are included as benchmarks [25]. A broader comparison with 2D and 3D nitrate‐based rare‐earth double perovskites is summarized in Table S5, where **3** lies at the upper end of the reported transition‐temperature range. Thermogravimetric analysis further confirms that **3** and **4** remain thermally stable well above their respective transition regions (Figure S6).

All four compounds exhibit detectable second‐harmonic generation (SHG) responses at 293 K, with intensities of ca. 0.10–0.14 times that of KDP, consistent with their non‐centrosymmetric ground states (Figure 3d). Variable‐temperature SHG measurements on **3** directly track the symmetry evolution across the two‐step transition (Figure 3e): upon heating, the SHG signal decreases sharply at the *C*2 → *R*32 transition, marking the loss of macroscopic polar order, and weakens further upon entering the high‐symmetry *F*432 phase; upon cooling, the signal recovers, demonstrating reversible symmetry switching within a single thermal cycle.

The real part of the complex permittivity, *ε′*, of **3** reproduces the two thermal anomalies as two distinct dielectric features (Figure 3f). The lower‐temperature anomaly is broad and strongly frequency‐dependent: between 5 kHz and 2 MHz, the *ε*′ maximum shifts to higher temperature, suggestive of relaxor‐like dielectric behavior reminiscent of PbMg_1/3_Nb_2/3_O_3_ (PMN) and related (RM3HQ)_2_RbLn(NO_3_)_6_ systems (Ln = La, Ce, Pr, Eu) [25, 26, 27, 28, 60]. In contrast, the higher‐temperature anomaly is sharper and nearly frequency‐independent, consistent with the second thermal anomaly and the further suppression of the SHG signal at the *R*32 → *F*432 step.

The ferroelectricity of **3** was verified by polarization–electric field (*P–E*) measurements at 293 K and 50 Hz, which yielded a closed hysteresis loop with *P*_s_ ≈ 1.4 µC cm^−2^ and *E*_c_ ≈ 17 kV cm^−1^ (Figure 3g). The *C*2 → *R*32 transition corresponds to the Aizu species 32*F*2 [61], permitting three symmetry‐equivalent polarization directions in the polar phase. The *P*_s_ value is substantially larger than that of the isostructural RbLa analogue, (RM3HQ)_2_RbLa(NO_3_)_6_ (∼0.18 µC cm^−2^) [25], although it remains relatively small compared with those of many recently reported molecular ferroelectrics [62, 63, 64, 65, 66, 67, 68, 69, 70].

Quasi‐static *d*_33_ measurements on independent single crystals of **3**, under the measurement conditions described in Figure S9, yielded an average |*d*_33_| of ca. 56 pC N^−1^ (Figure 3h). Benchmarking against representative molecular ferroelectrics and piezoelectrics (Figure 3i, with an extended comparison in Table S6) shows that **3** exhibits appreciable macroscopic piezoelectricity. Although its *d*_33_ is lower than that of TMCM‐MnCl_3_ (185 pC N^−1^) [3], it exceeds those of several widely studied molecular and polymeric piezoelectrics, including [(CF_3_‐C_6_H_4_‐NH_3_)(18‐crown‐6)][TFSA] (42 pC N^−1^) [52], PVDF (28 pC N^−1^) [56], and TGS (20 pC N^−1^) [59]. Notably, relative to the RbEu analogue (RM3HQ)_2_RbEu(NO_3_)_6_ (103 pC N^−1^) [28], compound **3** retains appreciable piezoelectricity (56 pC N^−1^) while raising the polar‐to‐nonpolar transition temperature to 414 K, far above room temperature.

### 2.4 Ferroelectric and Ferroelastic Domain Behavior of 3

Piezoresponse force microscopy (PFM) measurements were performed on a thin‐film sample of **3** to investigate its nanoscale ferroelectric domain structure (Figure 4a–f). The topography image shows a smooth surface (Figure 4a). Vertical PFM amplitude and phase images reveal stripe‐like ferroelectric domains, with opposite phase contrasts between adjacent stripes indicating oppositely oriented out‐of‐plane polarization components (Figure 4b,c). Local switching spectroscopy at a selected point produced a butterfly‐shaped amplitude–voltage loop and a phase–voltage hysteresis loop (Figure 4d), demonstrating reversible polarization switching at the nanoscale. Lateral PFM amplitude and phase images resolved in‐plane domain contrast (Figure 4e,f); the coexistence of vertical and lateral piezoresponses is consistent with the multiaxial nature expected for the Aizu species 32*F*2 [61].

**FIGURE 4:**
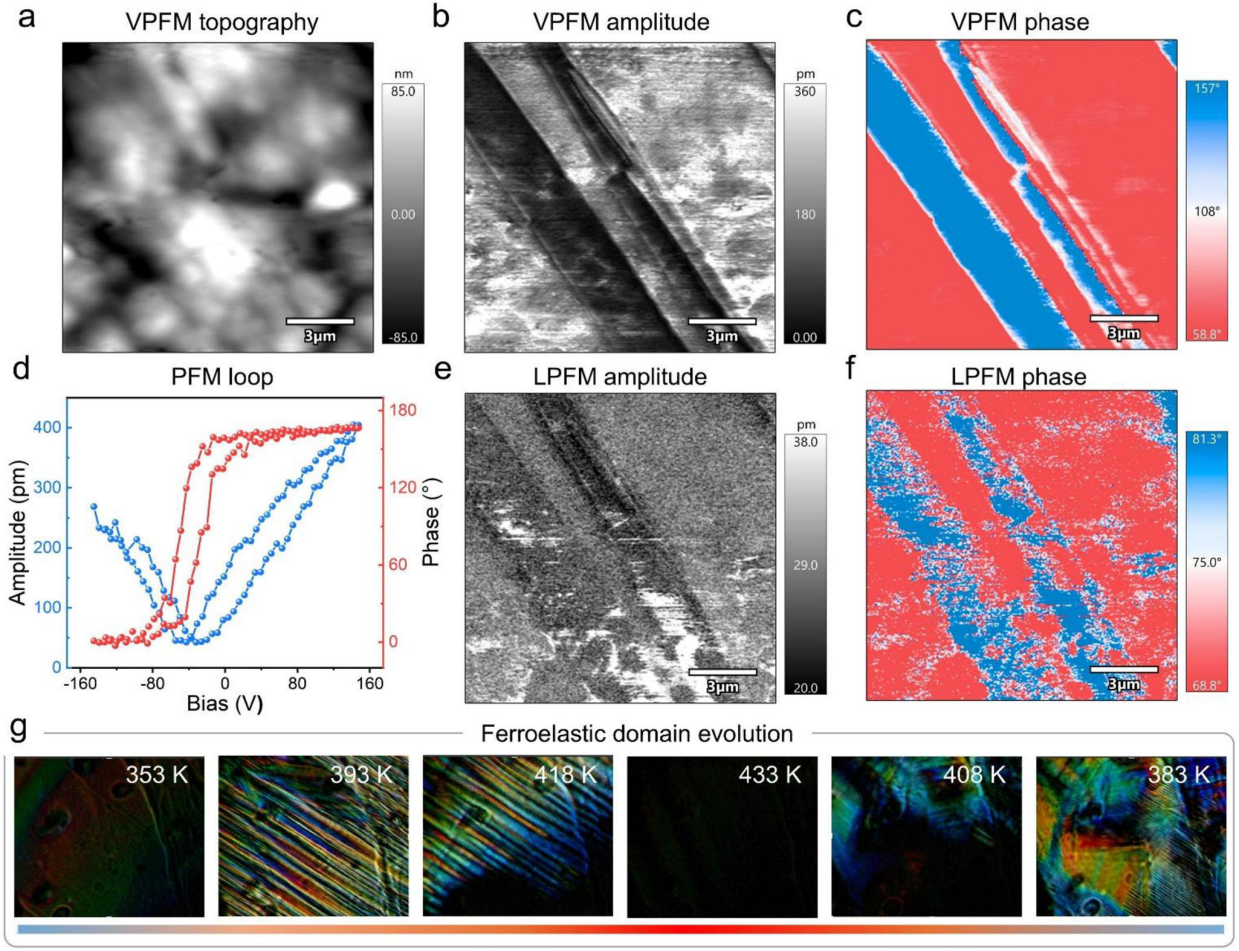
Ferroelectric and ferroelastic domain behavior of compound **3**. (a) PFM topography image of a thin film of **3**. (b) Vertical PFM amplitude image. (c) Vertical PFM phase image. (d) Amplitude–voltage butterfly loop and phase–voltage hysteresis loop. (e) Lateral PFM amplitude image. (f) Lateral PFM phase image. (g) Variable‐temperature polarized‐light microscopy images of ferroelastic domain evolution in **3**, collected during heating from 353 to 433 K and during subsequent cooling from 433 to 383 K.

Variable‐temperature polarized‐light microscopy was used to track the ferroelastic domain evolution across the phase‐transition region (Figure 4g). The *C*2 → *R*32 transition corresponds to the Aizu species 32*F*2, and the *R*32 → *F*432 transition corresponds to 432*F*32 [61]. Upon heating, stripe‐like ferroelastic textures became clearly visible at 393 K, faded by 418 K, and vanished at 433 K as the crystal enters the high‐symmetry phase. Upon cooling, the textures reappeared at 408 K and further developed at 383 K. The reversible appearance and disappearance of these textures are consistent with the ferroelastic domain evolution associated with the transitions. The complete evolution is shown in Movie S1.

### 2.5 Temperature‐Dependent Eu^3^^+^ Luminescence across Phase Transitions

The order–disorder transitions discussed above also perturb the local coordination environment around Eu^3+^ centers. Because nitrate groups both coordinate Eu^3+^ and connect neighboring Eu(NO_3_)_6_ units, changes in nitrate disorder and *B* ^I^‐site dynamics can modify the Eu‐centered ligand field. Therefore, Eu^3+^ luminescence provides an optical probe of local ligand‐field changes around Eu(NO_3_)_6_ [46, 71, 72]. At 293 K, the absorption, excitation, and emission spectra of compounds **1–4** are shown in Figure 5a. Their absorption spectra are similar, with comparable estimated optical bandgaps (Figure S10). Upon excitation at 397 nm, corresponding to the ^7^F_0_ → ^5^L_6_ transition, all four compounds exhibit typical Eu^3+^ red emission, including the ^5^D_0_ → ^7^F_1_ magnetic‐dipole band near 592 nm, the electric‐dipole ^5^D_0_ → ^7^F_2_ band near 616 nm, and a weaker ^5^D_0_ → ^7^F_4_ band near 689 nm [46, 47]. Under 365 nm excitation, the PLQYs of **1–4** are 12.4%, 7.8%, 18.2%, and 7.0%, respectively (Figure S11).

**FIGURE 5:**
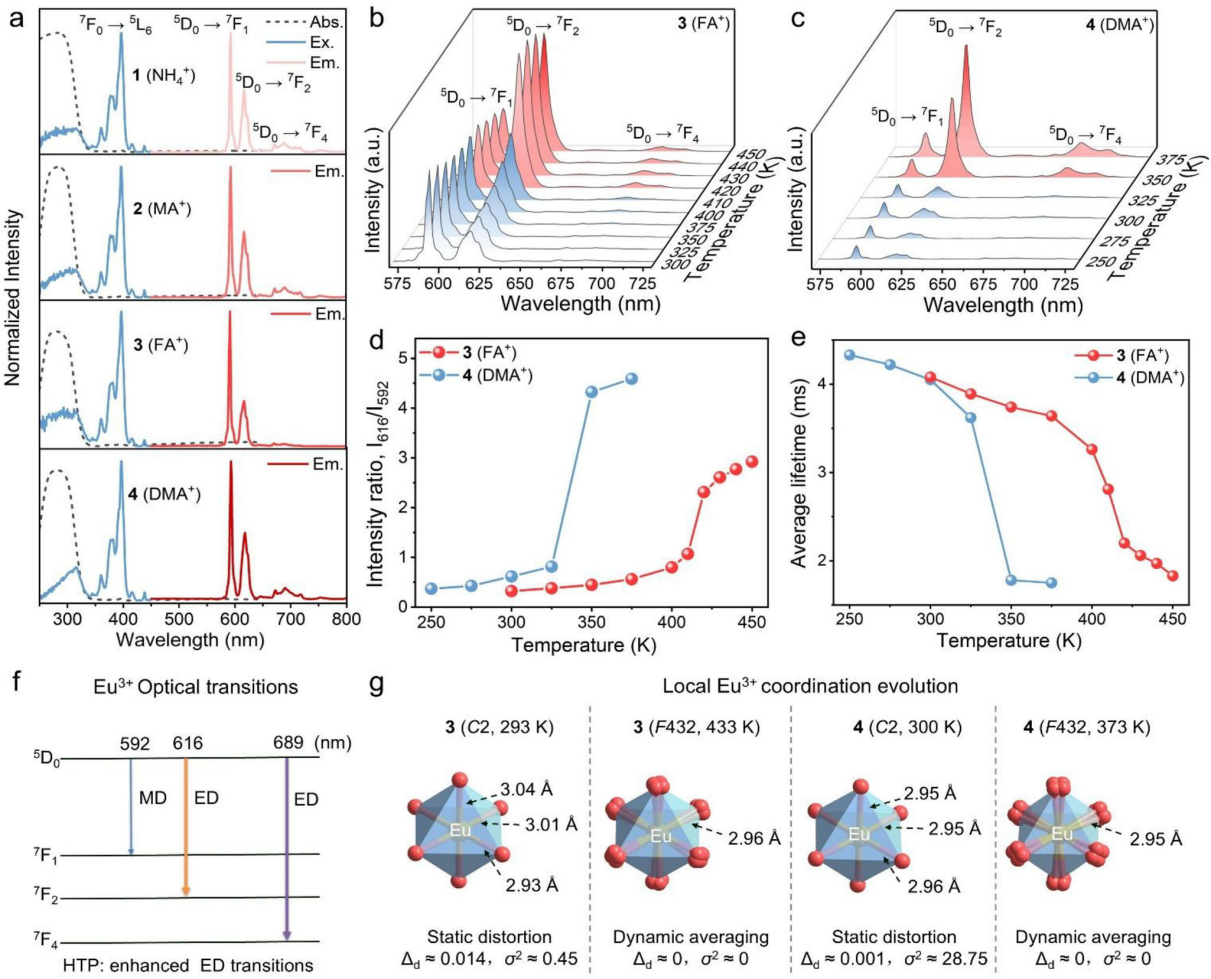
(a) Room‐temperature absorption (Abs.), excitation (Ex.), and emission (Em.) spectra of compounds **1–4**. (b,c) Temperature‐dependent Eu^3+^ emission spectra of **3** (300–450 K) and **4** (250–375 K) across their phase‐transition regions. (d) Temperature‐dependent I_616_/I_592_ ratios for **3** (red) and **4** (blue); I_616_ and I_592_ are the electric‐dipole (ED, 616 nm) and magnetic‐dipole (MD, 592 nm) emission intensities. (e) Temperature‐dependent average Eu^3+^ emission lifetimes for **3** (red) and **4** (blue). (f) Schematic Eu^3+^ optical transitions, highlighting enhanced ED emission in the HTP. (g) Local Eu^3+^ coordination evolution of **3** and **4** from *C*2 to *F*432, with NO_3_^−^ groups represented by centroids. Eu···NO_3_‐centroid distances, centroid‐distance distortion indices Δ*_d_*, and angular variances *σ* ^2^ are annotated.

Temperature‐dependent emission spectra reveal a clear redistribution of Eu^3+^ emission channels during the phase transitions. For compound **3**, upon heating from 300 to 450 K across the transition region, the ^5^D_0_ → ^7^F_1_ magnetic‐dipole band remains nearly unchanged in relative intensity, whereas the ^5^D_0_ → ^7^F_2_ and ^5^D_0_ → ^7^F_4_ electric‐dipole bands are markedly enhanced (Figure 5b). Compound **4** shows the same trend over 250–375 K (Figure 5c). Accordingly, the 616/592 nm emission intensity ratio I_616_/I_592_ increases from 0.32 to 2.92 for **3** and from 0.37 to 4.59 for **4** (Figure 5d), with the larger change in **4** indicating a more pronounced change in the relative intensities of the two emission channels [46, 71]. Consistent with these spectral changes, the average Eu^3+^ emission lifetimes decrease with increasing temperature and drop noticeably near the corresponding transition regions (Figure 5e), indicating enhanced nonradiative relaxation during the phase transitions [47, 73, 74].

The redistribution of Eu^3+^ emission channels can be understood from the different sensitivities of these optical transitions to the local ligand field (Figure 5f). The ^5^D_0_ → ^7^F_1_ magnetic‐dipole transition is relatively insensitive to the ligand environment and can therefore serve as an internal reference. By comparison, the ^5^D_0_ → ^7^F_2_ transition is a hypersensitive transition that is strongly affected by local asymmetry around Eu^3+^ [46, 71, 73]. Thus, the increase in I_616_/I_592_ is consistent with changes in the relative emission contributions of the two channels during the phase transitions, which may arise from modifications of the local Eu^3+^ environment.

Local structural analysis helps rationalize the different ligand‐field responses of compounds **3** and **4** (Figure 5g). The Eu(NO_3_)_6_ geometries were evaluated using Eu···NO_3_‐centroid distances, a centroid‐distance distortion index Δ*_d_*, and an angular variance *σ* ^2^, with NO_3_^−^ groups represented by their centroids. In the *C*2 phase of **3**, the Eu···NO_3_‐centroid distances span ∼2.93–3.04 Å, giving Δ*_d_* ≈ 0.014 and *σ* ^2^ ≈ 0.45, indicating weak static distortion mainly reflected in distance dispersion. In the *C*2 phase of **4**, the corresponding distances are nearly identical, ∼2.95–2.96 Å, with Δ*_d_* ≈ 0.001 but *σ* ^2^ ≈ 28.75, indicating a larger angular contribution to the local distortion. Upon transformation to *F*432, the crystallographic models of both compounds become dynamically averaged, giving regular average Eu(NO_3_)_6_ geometries with Δ*_d_* ≈ 0 and *σ* ^2^ ≈ 0. This crystallographic regularity, however, arises from extensive nitrate disorder rather than from a truly static, ordered Eu(NO_3_)_6_ environment. The greater spatial dispersion of nitrate O atoms around Eu in **4** than in **3** (Figure S14) suggests differences in their local coordination environments, which may contribute to the different temperature‐dependent I_616_/I_592_ responses. During the structural transitions, changes in the local coordination environment around Eu^3+^ may alter the relative intensities of the 616 and 592 nm emission channels, thereby providing an optical response to the phase transitions.

The increase in I_616_/I_592_ is notable because the average crystallographic symmetry increases from *C*2 to *F*432. Under this higher average symmetry, the ^5^D_0_ → ^7^F_2_ electric‐dipole transition, which is hypersensitive to local asymmetry around Eu^3+^, would normally be expected to weaken rather than strengthen [46, 73]. This apparent discrepancy suggests that Eu^3+^ emission reflects local ligand‐field asymmetry beyond the average crystallographic structure. The enhanced electric‐dipole emission is therefore consistent with locally low‐symmetry Eu(NO_3_)_6_ environments associated with dynamically disordered *B* ^I^‐site cations, NO_3_^−^ groups, and RM3HQ^+^ cations, which are averaged in diffraction but remain detectable by Eu^3+^ luminescence [46, 71, 73].

This behavior distinguishes the present compounds from conventional Eu^3+^‐based luminescent thermometers and Eu^3+^‐doped ferroic or phase‐transition materials, in which Eu^3+^ is introduced as an external optical probe [74, 75, 76, 77, 78]. Here, Eu^3+^ is an integral part of the nitrate‐based double‐perovskite structure. Therefore, the temperature‐dependent change in I_616_/I_592_ does not merely serve as a thermometric signal but reflects transition‐related changes in the local ligand field around Eu^3+^, providing a dopant‐free optical signature of the phase transitions.

## 3 Conclusion

In conclusion, molecular double perovskites offer crystallographically distinct sites for connecting local molecular interactions with extended connectivity, structural phase transitions, and coupled physical responses. This work demonstrates that, in nitrate‐based rare‐earth double perovskites, the *B* ^I^‐site can serve as a flexible handle, where *B* ^I^···NO_3_ interactions are closely linked to nitrate connectivity and Eu‐centered ligand‐field variations. Replacing smaller NH_4_^+^/MA^+^ with larger FA^+^/DMA^+^ at the *B* ^I^‐site converts mixed corner‐/face‐sharing non‐perovskite structures into 3D corner‐sharing double perovskites and raises the ferroelectric transition temperature as high as 414 K. In compound **3**, this elevated‐temperature transition is accompanied by ferroelectric hysteresis, reversible ferroelastic domains, and macroscopic piezoelectricity, confirming multiaxial ferroic behavior. In addition, the temperature‐dependent change in the PL intensity ratio (I_616_/I_592_) reflects transition‐related changes in the local ligand field of Eu^3+^, providing a luminescence signature of the phase transitions. These results diversify the family of molecular perovskites and demonstrate clear structure–function relations in hybrid ferroic materials.

## Author Contributions

**Le Ye**: conceptualization, methodology, investigation, formal analysis, data curation, visualization, and Writing – original draft. **Guang‐Yuan Hu**: investigation and validation. **Yarou Duan**: investigation and validation. **Yutong Lin**: visualization and validation. **Ding‐Chong Han**: investigation and validation. **Zi‐Luo Fang**: investigation and validation. **Liang‐Han Shen**: investigation and validation. **Zhe Dong**: supervision and writing – review and editing. **Wei‐Xiong Zhang**: supervision and writing – review and editing. **Na Wang**: investigation, funding acquisition, and writing – review and editing. **Le‐Ping Miao**: investigation, funding acquisition, supervision, and writing – review and editing. **Heng‐Yun Ye**: conceptualization, funding acquisition, supervision, and writing – review and editing. **Lingling Mao**: conceptualization, funding acquisition, supervision, project administration, resources, writing – original draft, and writing – review and editing. All authors discussed the results and approved the final manuscript.

## Conflicts of Interest

The authors declare no conflicts of interest.

## Supporting information




**Supporting File 1**: advs78404‐sup‐0001‐SuppMat.docx.

**Supporting File 2**: advs78404‐sup‐0002‐MovieS1.mp4.

## Data Availability

The data that support the findings of this study are available in the Supporting Information of this article. The Supporting Information contains experimental procedures, crystallographic data and refinements, supplementary structural and physical characterization, photophysical analyses, equations, figures, tables, and Movie S1 showing the temperature‐dependent ferroelastic domain evolution of compound **3**. Deposition Numbers 2529489 (for **1** at 293 K), 2529490 (for **2** at 100 K), 2529492 (for **3** at 293 K), 2529493 (for **3** at 420 K), 2529494 (for **3** at 433 K), 2529491 (for **4** at 300 K), 2553218 (for **4** at 353 K), and 2553219 (for **4** at 373 K) contain the supplementary crystallographic data for this paper. These data are provided free of charge by the joint Cambridge Crystallographic Data Centre and Fachinformationszentrum Karlsruhe Access Structures service (https://www.ccdc.cam.ac.uk/structures/).
