# Supplementary material for: Molecularly Engineered Nitrate Double Perovskites with Elevated Ferroelectric Phase Transition Temperature and Large Piezoelectric Response
Source: Adv Sci (Weinh). 2026 Oct 26:e78404. Online ahead of print. doi: 10.1002/advs.78404 (PMC13698014; doi:10.1002/advs.78404)
Supplement: Supplementary file 1 [file ADVS-9999-e78404-s002.docx]

*Supporting Information*

Molecularly Engineered Nitrate Double Perovskites with Elevated Ferroelectric Phase Transition Temperature and Large Piezoelectric Response

Le Ye^1,2^, Guang-Yuan Hu^3^, Yarou Duan^1,2^, Yutong Lin^1,2^, Ding-Chong Han^4^, Zi-Luo Fang^4^, Zhe Dong^1^, Wei-Xiong Zhang^4^, Liang-Han Shen^3^, Na Wang^3^, Le-Ping Miao^3^*, Heng-Yun Ye^3^*, and Lingling Mao^1,2^*

^1^*Department of Chemistry, Southern University of Science and Technology, Shenzhen 518055, China.*

^2^*SUSTech Energy Institute for Carbon Neutrality, Southern University of Science and Technology, Shenzhen 518055, China.*

^3^*Chaotic Matter Science Research Center, Jiangxi Province Key Laboratory of Functional Crystalline Materials Chemistry, International Institute for Innovation, Jiangxi University of Science and Technology, Ganzhou 341000, China.*

^4^*MOE Key Laboratory of Bioinorganic and Synthetic Chemistry, IGCME, School of Chemistry, Sun Yat-sen University, Guangzhou 510275, China.*

*Corresponding authors

E-mail:

(L.M.) maoll@sustech.edu.cn;

(H.-Y.Y.) hyye@seu.edu.cn;

(L.-P.M.) miaoleping@jxust.edu.cn

*Table of Contents*

[Section S1. Experimental Section	S1](#_Toc231476570)

[Section S2. Supplementary Tables and Figures	S7](#_Toc231476571)

[Section S3. Supplementary Equations	S20](#_Toc231476572)

# Section S1. Experimental Section

**Materials**

(*R*)-3-hydroxyquinuclidine (R3HQ, 98%, Macklin), methyl iodide (CH_3_I, 99%, Macklin), europium nitrate hexahydrate (Eu(NO_3_)_3_·6H_2_O, 99.9%, Aladdin), silver carbonate (Ag_2_CO_3_, 98%, Aladdin), nitric acid (HNO_3_, 65–68%, Guangzhou Chemical Reagent Factory), ammonium chloride (NH_4_Cl, 99.9%, Macklin), methylammonium hydrochloride (MACl, 99.9%, Bidepharm), formamidinium hydrochloride (FACl, 98%, Bidepharm), dimethylammonium hydrochloride (DMACl, 98%, Bidepharm), and isopropanol (analytical grade, Aladdin) were purchased from commercial suppliers. Deionized water was prepared in-house. All reagents were used as received without further purification.

**Synthesis of RM3HQNO_3_**

R3HQ (10 mmol) and CH_3_I (12 mmol) were dissolved in isopropanol (30 mL) and refluxed at 80 °C for 12 h. After cooling to room temperature, the solvent was removed by rotary evaporation to afford (*R*)-*N*-methyl-3-hydroxyquinuclidinium iodide (RM3HQI) as a colorless solid, which was washed with diethyl ether and dried under vacuum overnight. The obtained RM3HQI was dissolved in deionized water (20 mL), and Ag_2_CO_3_ (6 mmol, slight excess relative to the 5 mmol required for complete I⁻ removal) was added portionwise to fully precipitate the iodide as AgI. The mixture was stirred at room temperature for 2 h and filtered under reduced pressure to remove the AgI precipitate. The resulting filtrate, containing the RM3HQ^+^ carbonate/bicarbonate salt, was then acidified by dropwise addition of dilute HNO_3_ under stirring; the carbonate was thereby neutralized with release of CO_2_, affording a clear, colorless RM3HQNO_3_ aqueous solution, whose concentration was taken as the molar amount of RM3HQI charged, assuming quantitative anion exchange. This solution was used directly in the subsequent crystallization step.

**Synthesis of (RM3HQ)_2_*B*^I^Eu(NO_3_)_6_ (*B*^I^ = NH_4_^+^, MA^+^, FA^+^, DMA^+^)**

An aqueous solution (10 mL) containing RM3HQNO_3_ (4 mmol), the corresponding *B*^I^-site hydrochloride salt (2 mmol, NH_4_Cl for **1**, MACl for **2**, FACl for **3**, and DMACl for **4**), Eu(NO_3_)_3_·6H_2_O (2 mmol), and dilute HNO_3_ (2 mmol) was prepared and subjected to slow evaporation on a heating stage at 40 °C for approximately 48 h, affording colorless block-shaped crystals of (RM3HQ)_2_(NH_4_)Eu(NO_3_)_6_ (**1**), (RM3HQ)_2_(MA)Eu(NO_3_)_6_ (**2**), (RM3HQ)_2_(FA)Eu(NO_3_)_6_ (**3**) and (RM3HQ)_2_(DMA)Eu(NO_3_)_6_ (**4**), respectively. For compound **3**, large single crystals suitable for physical property measurements were grown by slow evaporation in a desiccator charged with silica gel at room temperature over approximately two weeks. RM3HQNO_3_ was prepared on a scaled-up basis following the procedure described above. The crystallization solution (20 mL) contained RM3HQNO_3_ (20 mmol), FACl (10 mmol), Eu(NO_3_)_3_·6H₂O (10 mmol), and dilute HNO_3_ (10 mmol); small crystals obtained from the standard procedure above were added as seeds.

**Single-Crystal X-ray Diffraction (SCXRD)**

Data for compound **1** were collected at 293 K on a Bruker D8 Venture diffractometer using Ga Kα radiation (*λ* = 1.34139 Å). Data for compound **2** were collected at 100 K on a Rigaku ROD Synergy Custom diffractometer using Cu Kα radiation (*λ* = 1.54184 Å); the low temperature was used to obtain high-quality data, as crystals of **2** showed poor diffraction quality at room temperature. Data for compound **3** were collected at 293 K (RTP), 420 K (ITP), and 433 K (HTP) on a Rigaku XtaLAB Synergy R diffractometer, using Mo Kα radiation (*λ* = 0.71073 Å) for the RTP and HTP phases and Cu Kα radiation (*λ* = 1.54178 Å) for the ITP. For compound **4**, data were collected at 300 K (RTP), 353 K (ITP), and 373 K (HTP) on a Rigaku XtaLAB Synergy R diffractometer using Mo Kα radiation (*λ* = 0.71073 Å), to characterize the distinct crystallographic phases across the phase transitions. Data collected on the Bruker instrument were integrated with SAINT and corrected for absorption using SADABS (APEX3).[1] Data collected on the Rigaku instruments were processed with CrysAlisPro, with multi-scan absorption correction applied using SCALE3 ABSPACK.[2] Space groups were assigned based on systematic absences and intensity statistics and further verified using PLATON.[3] All structures were solved by intrinsic phasing (SHELXT) and refined by full-matrix least-squares on *F*^2^ (SHELXL) in OLEX2.[4-6] All non-hydrogen atoms were refined anisotropically. Hydrogen atoms bonded to carbon were placed in idealized positions and refined using a riding model, with *U*_iso_(H) = 1.2*U*_eq_(C) for CH and CH_2_ groups and 1.5Ueq(C) for CH_3_ groups. Hydroxyl O–H hydrogen atoms were located from difference Fourier maps and refined using a riding model with *U*_iso_(H) = 1.5*U*_eq_(O). N–H hydrogen atoms of the RM3HQ^+^ and *B*^I^-site cations were placed in geometrically idealized positions and refined using a riding model with *U*_iso_(H) = 1.2*U*_eq_(N); for the orientationally disordered *B*^I^-site cations, N–H hydrogen atoms were included in calculated positions where the disorder model permitted. Crystal data and structure refinement parameters are summarized in Tables S1–S3. The relatively large R values for the high-temperature phases are mainly associated with severe orientational/positional disorder and weakened diffraction at elevated temperatures. Crystallographic data have been deposited with the Cambridge Crystallographic Data Centre: CCDC 2529489 (**1**), 2529490 (**2**), 2529492 (**3**, RTP), 2529493 (**3**, ITP), 2529494 (**3**, HTP), 2529491 (**4**, RTP), 2553218 (**4**, ITP), and 2553219 (**4**, HTP), and are available free of charge from the CCDC website.

**Powder X-ray Diffraction (PXRD)**

PXRD patterns were collected on a Rigaku SmartLab diffractometer with Cu Kα radiation (*λ* = 1.54056 Å) operated at 45 kV and 200 mA. Data were recorded over 2*θ* = 5–50° at room temperature (step size = 0.02°, scan rate = 5° min⁻^1^) to assess phase purity.

**Optical characterization**

UV–vis diffuse reflectance spectra were recorded on a Shimadzu UV-3600i Plus spectrophotometer using BaSO_4_ as the reference standard. The reflectance data were converted to pseudo-absorbance using the Kubelka–Munk function, *F*(*R*) = (1 – *R*)^2^ / 2*R*, where *R* is the reflectance.[7] Steady-state photoluminescence (PL) and photoluminescence excitation (PLE) spectra were recorded at room temperature on an Edinburgh Instruments FLS1000 spectrometer equipped with a 450 W xenon lamp. Temperature-dependent PL spectra and time-resolved PL (TRPL) decay curves were collected on the same spectrometer equipped with a cryostat. For compound **3**, temperature-dependent measurements were performed from 300 to 400 K at 25 K intervals and from 400 to 450 K at 10 K intervals; for compound **4**, from 250 to 375 K at 25 K intervals. These temperature ranges were chosen to span the respective phase-transition regions. Compounds **1** and **2** were measured only at room temperature. TRPL decay curves were recorded using a microsecond flash lamp as the excitation source, monitoring the ^5^D_0_ → ^7^F_2_ emission of Eu^3+^ at ~616 nm. The decay curves were fitted with mono- or biexponential functions.[8] For biexponential decays, the average lifetime (*τ*_ave_) was calculated as the amplitude-weighted average: *τ*_ave_ = (A_1_*τ*_1_ + A_2_*τ*_2_) / (A_1_ + A_2_), where A_1_ and A_2_ are the fitted pre-exponential amplitudes and *τ*_1_ and *τ*_2_ are the corresponding lifetimes. Photoluminescence quantum yields (PLQYs) were measured at room temperature using an integrating sphere on a Xi Pu Guang Dian XPQY-EQE-Adv system. All samples were excited at 365 nm, and PLQY values were obtained by integrating the emission over the full spectral window of each sample.

**Thermal Analysis**

Thermogravimetric analysis (TGA) of compounds **3** and **4** was carried out on a TA Q50 system under nitrogen atmosphere at a heating rate of 10 °C min^-1^ from room temperature to 800 °C. Differential scanning calorimetry (DSC) measurements were conducted on powder samples of compounds **3** and **4** using a TA DSC Q2000 instrument over both heating and cooling cycles at a rate of 10 K min^-1^; the temperature was scanned from 293 K to 460 K for **3** and from 293 K to 380 K for **4**.

**Dielectric Measurements**

Variable-temperature dielectric measurements of **3** and **4** were performed on a TH2838A impedance analyzer at frequencies ranging from 5 kHz to 2 MHz with an applied voltage of 1.0 V. The temperature was scanned from 293 to 440 K for **3** and from 250 to 380 K for **4**, at a rate of ~4 K min^-1^ using an Oxford Instruments Mercury iTC controller. Powder samples were finely ground and pressed into pellets under ~5 GPa, with two magnetic sheets as electrodes.

**Polarization–Electric Field (*P*−*E*) Hysteresis Loop Measurements**

*P*−*E* hysteresis loops of **3** were measured along the polar [010] direction of the RTP using a single-crystal plate that was oriented, cut, and polished accordingly (thickness 0.5 mm, area 6 mm^2^). Crystal orientation was determined using a Rigaku Synergy diffractometer, and silver paste electrodes were applied to both surfaces. Measurements were performed using a Radiant Precision Premier II instrument on a Sawyer−Tower circuit at 293 K and 50 Hz.[9]

**Second Harmonic Generation (SHG) Measurements**

Powder SHG measurements of compounds **1–4** were performed at room temperature using the Kurtz–Perry method with a pulsed Nd:YAG laser (1064 nm).[10] Variable-temperature SHG experiments of compounds **3** and **4** were performed using the same method, with **3** measured from room temperature to 463 K and **4** measured from room temperature to 390 K, both at a rate of ~4 K min^-1^, and encompassing both heating and cooling cycles. Relative SHG intensities were evaluated using KH_2_PO_4_ (KDP) as a reference.

**Piezoresponse Force Microscopy (PFM) Measurements**

Thin-film samples of **3** for PFM measurements were prepared by drop-casting a suspension of finely ground crystals onto a conductive substrate. The samples were then dried at 45 °C before measurement. Nanoscale piezoresponse imaging and local polarization switching spectroscopy were conducted using a resonance-enhanced piezoresponse force microscope (Oxford Instruments, MFP-3D).[11] Domain imaging and polarization switching were performed using conductive Pt/Ir-coated silicon probes (EFM-50, Nanoworld). An AC driving voltage of 5 V was applied to the conductive tip at the second resonant peak of the cantilever-sample system to enhance sensitivity. Measurements were performed with the high-voltage package and in situ heating stage.

**Ferroelastic Domain Observations**

Ferroelastic domain structures of **3** were observed using an Olympus BX51TRF optical polarizing microscope equipped with an INSTEC HCC602 cooling/heating stage. The temperature was maintained with an accuracy of ±0.2 K during measurements.

# Section S2. Supplementary Tables and Figures

**Figure S1.** Photographs of compounds **1–4** in the (RM3HQ)_2_*B*^I^Eu(NO_3_)_6_ series: (**a**) **1** (*B*^I^ = NH_4_^+^), (b) **2** (*B*^I^ = MA^+^), (**c**) **3** (*B*^I^ = FA^+^), and (**d**) **4** (*B*^I^ = DMA^+^), recorded under daylight (top) and 365 nm UV irradiation (bottom).

**Figure S2.** PXRD patterns of compounds **1–4** in the (RM3HQ)_2_*B*^I^Eu(NO_3_)_6_ series: (**a**) **1** (*B*^I^ = NH_4_^+^), (b) **2** (*B*^I^ = MA^+^), (**c**) **3** (*B*^I^ = FA^+^), and (**d**) **4** (*B*^I^ = DMA^+^). Experimental patterns are shown in blue, and simulated patterns calculated from SCXRD data are shown in black.

**Table S1.** Crystal data and structure refinements for compounds **1–2**.

| Compound | **1** | **2** |
| --- | --- | --- |
| Empirical formula | C_16_H_36_EuN_9_O_20_ | C_17_H_38_EuN_9_O_20_ |
| Formula weight | 826.50 | 840.52 |
| Temperature [K] | 293.00(10) | 100.00(10) |
| Crystal system | monoclinic | trigonal |
| Space group | *P*2_1_ | *P*3_2_ |
| *a* [Å] | 10.1258(4) | 9.7616(2) |
| *b* [Å] | 16.1054(6) | 9.7616(2) |
| *c* [Å] | 18.7988(6) | 56.1864(16) |
| *α* [°] | 90 | 90 |
| *β* [°] | 91.779(2) | 90 |
| *γ* [°] | 90 | 120 |
| Volume [Å^3^] | 3064.23(19) | 4636.6(2) |
| *Z* | 4 | 6 |
| *ρ*_calc_ [gcm^−3^] | 1.792 | 1.806 |
| *μ* [mm^−1^] | 11.331 | 15.424 |
| *F*(000) | 1672 | 2556 |
| Radiation | Ga Kα (*λ* = 1.34139 Å) | Cu Kα (*λ* = 1.54184 Å) |
| Reflections collected | 40448 | 32431 |
| Independent reflections | 13332  *R*_int_ = 0.0488  *R*_sigma_ = 0.0482 | 9793  *R*_int_ = 0.1041  *R*_sigma_ = 0.0857 |
| Goodness-of-fit on *F*^2^ | 1.050 | 1.050 |
| Final *R* indexes [*I*≥2σ(*I*)] | *R*_1_ = 0.0582  w*R*_2_ = 0.1261 | *R*_1_ = 0.0821  w*R*_2_ = 0.2018 |
| Final *R* indexes [all data] | *R*_1_ = 0.0704  w*R*_2_ = 0.1342 | *R*_1_ = 0.0901  w*R*_2_ = 0.2081 |
| Flack X parameter | 0.270(9) | 0.019(9) |
| CCDC number | 2529489 | 2529490 |

*R* = ∑||*F*_o_|-|*F*_c_||/∑|*F*_o_|; *wR*_2_ = {∑*w*(|*F*_o_|^2^ - |*F*_c_|^2^)^2^/∑*w*(|*F*_o_|^2^)^2^}.^1/2^

Inversion twinning was refined using TWIN -1 0 0 0 -1 0 0 0 -1, giving BASF values of 0.270(9) and 0.019(9) for **1** at 293 K and **2** at 100 K, respectively. **Table S2.** Crystal data and structure refinements for compound **3** at 293, 420 and 433 K.

| Compound | **3** | | |
| --- | --- | --- | --- |
| Empirical formula | C_17_H_37_EuN_10_O_20_ | | |
| Formula weight | 853.52 | | |
| Temperature [K] | 293.00(10) | 420.0(2) | 433.00(10) |
| Phase | RTP | ITP | HTP |
| Crystal system | monoclinic | trigonal | cubic |
| Space group | *C*2 | *R*32 | *F*432 |
| *a* [Å] | 17.0059(15) | 10.0657(11) | 14.9549(6) |
| *b* [Å] | 9.6222(8) | 10.0657(11) | 14.9549(6) |
| *c* [Å] | 11.0805(10) | 28.336(6) | 14.9549(6) |
| *α* [°] | 90 | 90 | 90 |
| *β* [°] | 118.968(5) | 90 | 90 |
| *γ* [°] | 90 | 120 | 90 |
| Volume [Å^3^] | 1586.3(2) | 2486.3(8) | 3344.6(4) |
| *Z* | 2 | 3 | 4 |
| *ρ*_calc_ [gcm^−3^] | 1.787 | 1.710 | 1.695 |
| *μ* [mm^−1^] | 2.076 | 14.402 | 1.969 |
| *F*(000) | 864 | 1296 | 1728 |
| Radiation | Mo Kα (*λ* = 0.71073 Å) | Cu Kα (*λ* = 1.54178 Å) | Mo Kα (*λ* = 0.71073 Å) |
| Reflections collected | 13049 | 2410 | 1321 |
| Independent reflections | 4032  *R*_int_ = 0.0211  *R*_sigma_ = 0.0225 | 1018  *R*_int_ = 0.0629  *R*_sigma_ = 0.0596 | 262  *R*_int_ = 0.0441  *R*_sigma_ = 0.0222 |
| Goodness-of-fit on *F*^2^ | 1.049 | 1.010 | 1.050 |
| Final *R* indexes [*I*≥2σ(*I*)] | *R*_1_ = 0.0227  w*R*_2_ = 0.0577 | *R*_1_ = 0.0671  w*R*_2_ = 0.1660 | *R*_1_ = 0.1125  w*R*_2_ = 0.1476 |
| Final *R* indexes [all data] | *R*_1_ = 0.0227  w*R*_2_ = 0.0577 | *R*_1_ = 0.1153  w*R*_2_ = 0.2053 | *R*_1_ = 0.1468  w*R*_2_ = 0.1628 |
| Flack X parameter | 0.37(2) | 0.47(5) | 0.3(18) |
| CCDC number | 2529492 | 2529493 | 2529494 |

*R* = ∑||*F*_o_|-|*F*_c_||/∑|*F*_o_|; *wR*_2_ = {∑*w*(|*F*_o_|^2^ - |*F*_c_|^2^)^2^/∑*w*(|*F*_o_|^2^)^2^}.^1/2^

Inversion twinning was refined using the TWIN matrix −1 0 0 / 0 −1 0 / 0 0 −1, giving BASF values of 0.37(2) at 293 K, 0.47(5) at 420 K, and 0.30(18) at 433 K, respectively.

**Table S3.** Crystal data and structure refinements for compound **4** at 300, 353 and 373 K.

| Compound | **4** | | |
| --- | --- | --- | --- |
| Empirical formula | C_18_H_40_EuN_9_O_20_ | | |
| Formula weight | 854.55 | | |
| Temperature [K] | 300.00(10) | 353.00(10) | 373.00(10) |
| Phase | RTP | ITP | HTP |
| Crystal system | monoclinic | trigonal | cubic |
| Space group | *C*2 | *R*32 | *F*432 |
| *a* [Å] | 17.2635(6) | 10.1356(11) | 14.9808(6) |
| *b* [Å] | 9.8408(3) | 10.1356(11) | 14.9808(6) |
| *c* [Å] | 10.9001(3) | 28.039(6) | 14.9808(6) |
| *α* [°] | 90 | 90 | 90 |
| *β* [°] | 118.178(3) | 90 | 90 |
| *γ* [°] | 90 | 120 | 90 |
| Volume [Å^3^] | 1632.32(10) | 2494.6(8) | 3362.1(4) |
| *Z* | 2 | 3 | 4 |
| *ρ*_calc_ [gcm^−3^] | 1.739 | 1.706 | 1.688 |
| *μ* [mm^−1^] | 2.017 | 1.979 | 1.958 |
| *F*(000) | 868 | 1302 | 1736 |
| Radiation | Mo Kα (*λ* = 0.71073 Å) | Mo Kα (*λ* = 0.71073 Å) | Mo Kα (*λ* = 0.71073 Å) |
| Reflections collected | 12190 | 3691 | 6844 |
| Independent reflections | 4132  *R*_int_ = 0.0245  *R*_sigma_ = 0.0277 | 1332  *R*_int_ = 0.0462  *R*_sigma_ = 0.0651 | 432 *R*_int_ = 0.1554 *R*_sigma_ = 0.0581 |
| Goodness-of-fit on *F*^2^ | 1.103 | 0.992 | 1.051 |
| Final *R* indexes [*I*≥2σ(*I*)] | *R*_1_ = 0.0459  w*R*_2_ = 0.1148 | *R*_1_ = 0.0709 w*R*_2_ = 0.1682 | *R*_1_ = 0.0719 w*R*_2_ = 0.1096 |
| Final *R* indexes [all data] | *R*_1_ = 0.0467  w*R*_2_ = 0.1155 | *R*_1_ = 0.1477 w*R*_2_ = 0.2025 | *R*_1_ = 0.2261 w*R*_2_ = 0.1618 |
| Flack X parameter | 0.47(3) | 0.50(17) | 0.0(14) |
| CCDC number | 2529491 | 2553218 | 2553219 |

*R* = ∑||*F*_o_|-|*F*_c_||/∑|*F*_o_|; *wR*_2_ = {∑*w*(|*F*_o_|^2^ - |*F*_c_|^2^)^2^/∑*w*(|*F*_o_|^2^)^2^}.^1/2^

Inversion twinning was refined using the TWIN matrix −1 0 0 / 0 −1 0 / 0 0 −1, giving BASF values of 0.47(3) at 300 K, 0.50(17) at 353 K, and 0.0(14) at 373 K, respectively.

**Figure S3.** Crystal packing of **1** at 293 K viewed along (**a**) the *c*-axis and (**b**) the *a*-axis.

**Figure S4.** Crystal packing of **2** at 100 K viewed along (**a**) the *c*-axis and (**b**) the *b*-axis.

**Table S4.** Comparison of *B*^I^···Eu distances and framework topologies in (RM3HQ)_2_*B*^I^Eu(NO_3_)_6_ and related analogues.

| Compound | *B*^I^ | Space group | Avg. *B*^I^···Eu (Å) | Topology |
| --- | --- | --- | --- | --- |
| K analogue[12] | K^+^ | *P*2_1_ | 6.15 | Mixed corner-/face-sharing |
| Rb analogue[13] | Rb^+^ | *R*3 | 7.22 | Corner-sharing |
| **1** (this work) | NH_4_^+^ | *P*2_1_ | 6.33 | Mixed corner-/face-sharing |
| **2** (this work) | MA^+^ | *P*32 | 6.77 | Mixed corner-/face-sharing |
| **3** (this work) | FA^+^ | *C*2 | 7.42 | Corner-sharing |
| **4** (this work) | DMA^+^ | *C*2 | 7.52 | Corner-sharing |

**Figure S5.** Variable-temperature crystal structures of compound **4**. RTP, ITP, and HTP denote the room-temperature, intermediate-temperature, and high-temperature phases, respectively. (a–c) Crystal structures of the RTP (300 K, *C*2), ITP (353 K, *R*32), and HTP (373 K, *F*432) phases, with local *B*ᴵ(NO_3_)_6_ coordination environments and packing views shown for each phase. Red spheres denote nitrate groups; blue and pink octahedra denote *B*ᴵ(NO_3_)_6_ and Eu(NO_3_)_6_ units; cyan dashed lines indicate N–H···O hydrogen bonds. Atom colors: C, gray; H, white; N, blue; O, red.

**Table S5.** Comparison of compounds **3** and **4** with selected reported nitrate-bridged rare-earth molecular phase-transition materials.

| Compound | *B*^I^ site | Dimension | *T*_c_ / K | Ferroic behavior | Ref. |
| --- | --- | --- | --- | --- | --- |
| (R3HQ)_4_KCe(NO_3_)_8_ | K^+^ | 2D | 323 | Ferroelectric | [14] |
| (R3HQ)_4_NH_4_Ce(NO_3_)_8_ | NH_4_^+^ | 2D | 333 | Not reported | [14] |
| (R3HQ)_4_RbCe(NO_3_)_8_ | Rb^+^ | 2D | 350 | Not reported | [14] |
| (R3HQ)_4_RbEu(NO_3_)_8_ | Rb^+^ | 2D | 347 | Not reported | [14] |
| (R3HQ)_4_RbSm(NO_3_)_8_ | Rb^+^ | 2D | 350 | Not reported | [14] |
| (S3FQ)_4_RbEu(NO_3_)_8_ | Rb^+^ | 2D | 441 | Ferroelectric, Ferroelastic | [15] |
| (DMP)_2_CsLa(NO_3_)_6_ | Cs^+^ | 3D | 201 | Not reported | [16] |
| (ODMP)_2_CsLa(NO_3_)_6_ | Cs^+^ | 3D | 251 | Not reported | [16] |
| (TFDMP)_2_CsLa(NO_3_)_6_ | Cs^+^ | 3D | 307, 398 | Not reported | [16] |
| [(CH_3_)_3_NCH_2_Cl]_2_RbEu(NO_3_)_6_ | Rb^+^ | 3D | 259 | Not reported | [17] |
| [(CH_3_)_3_NCH_2_Br]_2_RbEu(NO_3_)_6_ | Rb^+^ | 3D | 281 | Not reported | [17] |
| [(CH_3_)_3_NCH_2_I]_2_RbEu(NO_3_)_6_ | Rb^+^ | 3D | 327, 405 | Not reported | [17] |
| (RM3HQ)_2_RbLa(NO_3_)_6_ | Rb^+^ | 3D | 278, 425 | Ferroelectric | [18] |
| (RM3HQ)_2_NH_4_La(NO_3_)_6_ | NH_4_^+^ | 3D | 294, 314, 403 | Not reported | [18] |
| (RM3HQ)_2_RbCe(NO_3_)_6_ | Rb^+^ | 3D | 280 | Ferroelectric | [19] |
| (RM3HQ)_2_RbPr(NO_3_)_6_ | Rb^+^ | 3D | 278 | Ferroelectric | [20] |
| (RM3HQ)_2_KEu(NO_3_)_6_ | K^+^ | 3D | 372 | Not reported | [12] |
| (RCM3HQ)_2_CsEu(NO_3_)_6_ | Cs^+^ | 3D | 344 | Ferroelectric, Ferroelastic | [21] |
| (RM3HQ)_2_(DMA)Eu(NO_3_)_6_ | DMA^+^ | 3D | 349, 353 | Not examined | This work (**4**) |
| (RM3HQ)_2_(FA)Eu(NO_3_)_6_ | FA^+^ | 3D | 414, 420 | Ferroelectric, Ferroelastic | This work (**3**) |
| (4FHQ)_2_RbEu(NO_3_)_6_ | Rb^+^ | 3D | 423 | Ferroelastic | [22] |

Abbreviations: R3HQ = (*R*)-3-hydroxyquinuclidinium; S3FQ = (*S*)-3-fluoroquinuclidinium; DMP = *N*,*N*-dimethylpyrrolidinium; ODMP = *N*,*N*-dimethyl-3-hydroxypyrrolidinium; TFDMP = *N*,*N*-dimethyl-3,3-difluoropyrrolidinium; [(CH_3_)_3_NCH_2_Cl]^+^ = chloromethyltrimethylammonium; [(CH_3_)_3_NCH_2_Br]^+^ = bromomethyltrimethylammonium; [(CH_3_)_3_NCH_2_I]^+^ = iodomethyltrimethylammonium; RM3HQ = (*R*)-*N*-methyl-3-hydroxyquinuclidinium; RCM3HQ = (*R*)-*N*-chloromethyl-3-hydroxyquinuclidinium; DMA = dimethylammonium; FA = formamidinium; 4FHQ = 4-fluoroquinuclidinium.

**Figure S6.** Thermogravimetric analysis of (**a**) **3** and (**b**) **4**.

**Figure S7.** Variable-temperature dielectric constant *ε*′ of **3** upon cooling, showing the reversibility of the dielectric anomalies.

**Figure S8.** (a) Variable-temperature SHG intensity of **4** upon heating and cooling from room temperature to 390 K, demonstrating the reversibility of the SHG response. (b) Variable-temperature dielectric constant (*ε*′) of **4** upon heating from 250 to 380 K at frequencies ranging from 5 kHz to 2 MHz.

**Figure S9.** Quasi-static *d*_33_ measurements on three independent single crystals of **3** (a–c), performed normal to their largest natural (110) faces. For each crystal, the photograph (top) and corresponding positive (middle) and negative (bottom) *d*_33_ readings are shown. The average |*d*_33_| is approximately 56 pC N^-1^.

**Table S6.** Comparison of the *d*_33_ value of compound **3** with selected reported molecular ferroelectrics and piezoelectrics.

| Compound | *d*_33_ (pC N^-1^) | Ref. |
| --- | --- | --- |
| (*R*-/*S*-α-PEA)_2_TeI_6_ | 2.6 | [23] |
| [(*S*)-NEA]_4_[Bi_2_Cl_10_] | 6.7 | [24] |
| Rochelle salt | 7 | [25] |
| γ-glycine | 10 | [26] |
| *R*-/*S*-MPCoCl_4_ | 10.5 | [27] |
| DIPAB | 11 | [28] |
| TGS | 20 | [29] |
| [(*R*)-3-hydroxy-pyrrolidinium]Sb_4_Br_17_ | 22 | [30] |
| *R*-(4MeOPEA)_2_SnBr_6_ | 22.27 | [31] |
| PVDF | 28 | [32] |
| (R-α-PEA)4Bi_2_I_10_ | 32 | [33] |
| (DMPA)3Bi_2_Br_9_ | 35 | [34] |
| (Cl-PA)_2_PbBr_4_ | 36 | [35] |
| [(CF_3_-C_6_H_4_-NH_3_)(18-crown-6)][TFSA] | 42 | [36] |
| *R*/*S*-4-BrPEAC | 49 | [37] |
| (RM3HQ)_2_(FA)Eu(NO_3_)_6_ | 56 | This work (**3**) |
| NDABCO-NH_4_-Br_3_ | 63 | [38] |
| (RM3HQ)_2_RbLa(NO_3_)_6_ | 106 | [18] |
| TMCM-MnCl_3_ | 185 | [39] |
| TMCM-CdCl_3_ | 383 | [29] |

Abbreviations: *R*-/*S-*α-PEA = *R*-/*S*-alpha-phenylethylammonium; (*S*)-NEA = (*S*)-naphthylethylammonium; MP = 2-methylpiperazinium; DIPAB = diisopropylammonium bromide; TGS = triglycine sulfate; 4MeOPEA = 4-methoxy-alpha-methylbenzylammonium; PVDF = poly(vinylidene fluoride); DMPA = *N*,*N*-dimethylisopropylammonium; Cl-PA = 3-chloropropylammonium; TFSA = bis(trifluoromethanesulfonyl)amide; 4-BrPEAC = 4-bromo-alpha-methylbenzylammonium chloride; NDABCO = *N*-amino-*N*'-diazabicyclo[2.2.2]octonium; RM3HQ = (*R*)-*N*-methyl-3-hydroxyquinuclidinium; TMCM = trimethylchloromethylammonium.

**Figure S10.** Tauc plots derived from the diffuse reflectance spectra of the (RM3HQ)_2_*B*^I^Eu(NO_3_)_6_ series: (a) **1** (NH_4_^+^), (b) **2** (MA^+^), (c) **3** (FA^+^), and (d) **4** (DMA^+^); the corresponding optical band gaps are 3.99, 4.02, 4.01, and 3.96 eV, respectively.

**Figure S11.** PLQYs of compounds **1–4** under 365 nm excitation: 12.4%, 7.8%, 18.2%, and 7.0%, respectively.

**Figure S12.** PL decay curves of (a) **3** and (b) **4** recorded during heating across their phase-transition regions. The temperature ranges are 300–450 K for **3** and 250–375 K for **4**. Lifetimes are listed in each panel.

**Figure S13.** Reversible PL response of compound **3** during the cooling process. (a) Temperature-dependent emission spectra of 3 collected from 450 to 300 K across the reversible phase-transition region. (b) Temperature-dependent I_616_/I_592_ intensity ratio. (c) Temperature-dependent PL decay curves, with the extracted lifetimes listed in the panel. (d) Temperature-dependent average lifetime during cooling.

**Figure S14.** Comparison of the local Eu-centered nitrate environments in the high-temperature phases of (a) **3** (433 K) and (b) **4** (373 K). The disordered nitrate O positions and corresponding Eu–O distances and O_1_···O_2_′ split-site separations are shown.

# Section S3. Supplementary Equations

The centroid-based distortion parameters used in this work were adapted from conventional octahedral bond-length distortion and bond-angle variance descriptors, with each NO_3_⁻ group represented by its centroid.[40,41]

**Eu···NO_3_-centroid distance distortion**

$$\Delta d=\frac{1}{6}\sum_{i=1}^{6} \left( \frac{d_{i}-d_{\text{avg}}}{d_{\text{avg}}} \right)^{2}$$

where *d_i_* denotes the individual Eu···NO_3_-centroid distance and *d*_avg_ is the average Eu···NO_3_-centroid distance within one Eu(NO_3_)_6_ unit.

**Angular variance of the Eu(NO_3_-centroid)_6_ geometry**

$$\sigma^{2}=\frac{1}{11}\sum_{i=1}^{12} \left( \alpha_{i} - {90}^{\circ} \right)^{2}$$

where *α_i_* denotes the *i*-th cis NO_3_-centroid···Eu···NO_3_-centroid angle, namely the angle formed between two neighboring Eu···NO_3_-centroid directions within one Eu(NO_3_)_6_ unit. For an ideal octahedral arrangement of six NO_3_⁻ centroids around Eu, all *α_i_* = 90°, and thus *σ*^2^ = 0.

**References**

1. Bruker, *APEX3, SAINT, and SADABS*, Bruker AXS Inc., Madison, WI, USA, (2016).

2. Rigaku Oxford Diffraction, *CrysAlisPro*, Rigaku Oxford Diffraction, Yarnton, UK, (2019).

3. A. L. Spek, “Single-Crystal Structure Validation with the Program PLATON,” *Journal of Applied Crystallography* 36 (2003): 7–13, <https://doi.org/10.1107/S0021889802022112>.

4. G. M. Sheldrick, “SHELXT – Integrated Space-Group and Crystal-Structure Determination,” *Acta Crystallographica Section A: Foundations and Advances* 71 (2015): 3–8, <https://doi.org/10.1107/S2053273314026370>.

5. G. M. Sheldrick, “Crystal Structure Refinement with SHELXL,” *Acta Crystallographica Section C: Structural Chemistry* 71 (2015): 3–8, <https://doi.org/10.1107/S2053229614024218>.

6. O. V. Dolomanov, L. J. Bourhis, R. J. Gildea, J. A. K. Howard, and H. Puschmann, “OLEX2: A Complete Structure Solution, Refinement and Analysis Program,” *Journal of Applied Crystallography* 42 (2009): 339–341, <https://doi.org/10.1107/S0021889808042726>.

7. P. Kubelka, “New Contributions to the Optics of Intensely Light-Scattering Materials. Part I,” *Journal of the Optical Society of America* 38 (1948): 448–457, <https://doi.org/10.1364/JOSA.38.000448>.

8. E. Fišerová and M. Kubala, “Mean Fluorescence Lifetime and Its Error,” *Journal of Luminescence* 132 (2012): 2059–2064, <https://doi.org/10.1016/j.jlumin.2012.03.038>.

9. C. B. Sawyer and C. H. Tower, “Rochelle Salt as a Dielectric,” *Physical Review* 35 (1930): 269–273, <https://doi.org/10.1103/PhysRev.35.269>.

10. S. K. Kurtz and T. T. Perry, “A Powder Technique for the Evaluation of Nonlinear Optical Materials,” *Journal of Applied Physics* 39 (1968): 3798–3813, <https://doi.org/10.1063/1.1656857>.

11. S. Jesse, A. P. Baddorf, and S. V. Kalinin, “Switching Spectroscopy Piezoresponse Force Microscopy of Ferroelectric Materials,” *Applied Physics Letters* 88 (2006): 062908, <https://doi.org/10.1063/1.2172216>.

12. Q.-H. Zou, Z.-J. Wang, L.-K. Wu, Y. Feng, L.-L. Jiang, J.-R. Li, H.-Y. Ye, and C. Shi, “Three-Dimensional Bimetallic Ammonium K–Eu Nitrate with a Rare (6,6)-Connected Ion Topology Exhibiting Structural Phase Transition and Photoluminescence Properties,” *Inorganic Chemistry* 63 (2024): 10817–10822, <https://doi.org/10.1021/acs.inorgchem.4c01520>.

13. C.-F. Wang, C. Shi, A. Zheng, Y. Wu, L. Ye, N. Wang, H.-Y. Ye, M.-G. Ju, P. Duan, J. Wang, and Y. Zhang, “Achieving Circularly Polarized Luminescence and Large Piezoelectric Response in Hybrid Rare-Earth Double Perovskite by a Chirality Induction Strategy,” *Materials Horizons* 9 (2022): 2450–2459, <https://doi.org/10.1039/D2MH00698G>.

14. C. Shi, L. Ye, Z.-X. Gong, J.-J. Ma, Q.-W. Wang, J.-Y. Jiang, M.-M. Hua, and H.-Y. Ye, “Two-Dimensional Organic–Inorganic Hybrid Rare-Earth Double Perovskite Ferroelectrics,” *Journal of the American Chemical Society* 142 (2020): 545–551, <https://doi.org/10.1021/jacs.9b11697>.

15. N. Wang, Z.-J. Xu, H.-F. Ni, W. Luo, H.-K. Li, M.-L. Ren, C. Shi, H.-Y. Ye, X.-B. Fu, Y. Zhang, and L.-P. Miao, “Molecular Engineering Regulation Achieving Out-of-Plane Polarization in Rare-Earth Hybrid Double Perovskites for Ferroelectrics and Circularly Polarized Luminescence,” *Angewandte Chemie International Edition* 63 (2024): e202409796, <https://doi.org/10.1002/anie.202409796>.

16. Y. Lei, G. Peng, Y. Xu, Z. Li, and Z. Jin, “Molecular Engineering Tunes Lattice Dynamics in Hybrid Lanthanide Pseudohalides for Superior X-ray Detection,” *Materials Today* 95 (2026): 103287, <https://doi.org/10.1016/j.mattod.2026.103287>.

17. H.-K. Li, L.-P. Wang, Z.-Z. Cui, Q. Xu, L.-L. Zou, N. Wang, L.-P. Miao, H.-Y. Ye, and C. Shi, “A Series of Bimetallic Ammonium RbEu Nitrates Exhibiting Switchable Dielectric Constant and Photoluminescence Properties,” *Journal of Materials Chemistry C* 12 (2024): 14122–14128, <https://doi.org/10.1039/D4TC01910E>.

18. C. Shi, J.-J. Ma, J.-Y. Jiang, M.-M. Hua, Q. Xu, H. Yu, Y. Zhang, and H.-Y. Ye, “Large Piezoelectric Response in Hybrid Rare-Earth Double Perovskite Relaxor Ferroelectrics,” *Journal of the American Chemical Society* 142 (2020): 9634–9641, <https://doi.org/10.1021/jacs.0c00480>.

19. Z.-B. Hu, C.-F. Wang, T.-T. Sha, C. Shi, L. Ye, H.-Y. Ye, Y. Song, Y.-M. You, and Y. Zhang, “An Effective Strategy of Introducing Chirality to Achieve Multifunctionality in Rare-Earth Double Perovskite Ferroelectrics,” *Small Methods* 6 (2022): 2200421, <https://doi.org/10.1002/smtd.202200421>.

20. Q.-Q. Jia, H.-F. Lu, J.-Q. Luo, Y.-Y. Zhang, H.-F. Ni, F.-W. Zhang, J. Wang, D.-W. Fu, C.-F. Wang, and Y. Zhang, “Organic–Inorganic Rare-Earth Double Perovskite Ferroelectric with Large Piezoelectric Response and Ferroelasticity for Flexible Composite Energy Harvesters,” *Small* 20 (2024): 2306989, <https://doi.org/10.1002/smll.202306989>.

21. C.-F. Wang, Y. Yang, Y. Hu, C. Ma, H.-F. Ni, P.-G. Liu, H.-F. Lu, Z.-X. Zhang, J. Wang, Y. Zhang, D.-W. Fu, K. Zhao, and Y. Zhang, “Exploring Aqueous Solution-Processed Pseudohalide Rare-Earth Double Perovskite Ferroelectrics toward X-Ray Detection with High Sensitivity,” *Angewandte Chemie International Edition* 63 (2024): e202413726, <https://doi.org/10.1002/anie.202413726>.

22. Q. Xu, L. Ye, R.-M. Liao, Z. An, C.-F. Wang, L.-P. Miao, C. Shi, H.-Y. Ye, and Y. Zhang, “H/F Substitution Induced Large Increase of Tc in a 3D Hybrid Rare-Earth Double Perovskite Multifunctional Compound,” *Chemistry – A European Journal* 28 (2022): e202200521, <https://doi.org/10.1002/chem.202200521>.

23. K. Tao, Q. Li, and Q. Yan, “Narrow-Bandgap Tellurium-Based Chiral Hybrid Perovskite Single Crystals,” *The Journal of Physical Chemistry Letters* 15 (2024): 6024–6030, <https://doi.org/10.1021/acs.jpclett.4c01262>.

24. S. K. Mahato, V. Kushwaha, J. K. Zaręba, and R. Boomishankar, “A Chiral Edge-Sharing Bioctahedral Chlorobismuthate(III) Hybrid Ferroelectric and Its Application in Piezoelectric Energy Harvesting,” *Inorganic Chemistry* 64 (2025): 21194–21203, <https://doi.org/10.1021/acs.inorgchem.5c03925>.

25. W.-Q. Liao, Y.-Y. Tang, P.-F. Li, Y.-M. You, and R.-G. Xiong, “Competitive Halogen Bond in the Molecular Ferroelectric with Large Piezoelectric Response,” *Journal of the American Chemical Society* 140 (2018): 3975–3980, <https://doi.org/10.1021/jacs.7b12524>.

26. R. Wang, J. Sui, and X. Wang, “Natural Piezoelectric Biomaterials: A Biocompatible and Sustainable Building Block for Biomedical Devices,” *ACS Nano* 16 (2022): 17708–17728, <https://doi.org/10.1021/acsnano.2c08164>.

27. B.-Y. Jian, X. Lv, L.-C. An, and G.-M. Wang, “Shear Piezoelectricity and Electromechanical Conversion for a Pair of Chiral Hybrid Metal Halides with Reversible Phase Transition,” *Small* 21 (2025): 2508584, <https://doi.org/10.1002/smll.202508584>.

28. D.-W. Fu, H.-L. Cai, Y. Liu, Q. Ye, W. Zhang, Y. Zhang, X.-Y. Chen, G. Giovannetti, M. Capone, J. Li, and R.-G. Xiong, “Diisopropylammonium Bromide Is a High-Temperature Molecular Ferroelectric Crystal,” *Science* 339 (2013): 425–428, <https://doi.org/10.1126/science.1229675>.

29. H.-P. Lv, W.-Q. Liao, Y.-M. You, and R.-G. Xiong, “Inch-Size Molecular Ferroelectric Crystal with a Large Electromechanical Coupling Factor on Par with Barium Titanate,” *Journal of the American Chemical Society* 144 (2022): 22325–22331, <https://doi.org/10.1021/jacs.2c11213>.

30. H.-Y. Shen, L. He, P.-P. Shi, and Q. Ye, “Lead-Free Organic–Inorganic Hybrid Semiconductors and NLO Switches Tuned by Dimensional Design,” *Journal of Materials Chemistry C* 9 (2021): 4338–4343, <https://doi.org/10.1039/D1TC00278C>.

31. H.-R. Yang, X.-Q. Wei, G.-Z. Wang, C. Zhao, S.-S. Huang, Z.-G. Li, and W. Li, “Enabling Energy Harvesting and Underwater Ultrasound Detection with Chiral 0D Hybrid Tin Bromides,” *Chinese Journal of Chemistry* 43 (2025): 3213–3220, <https://doi.org/10.1002/cjoc.70242>.

32. S. Mohammadpourfazeli, S. Arash, A. Ansari, S.-Y. Yang, K. Mallick, and R. Bagherzadeh, “Future Prospects and Recent Developments of Polyvinylidene Fluoride (PVDF) Piezoelectric Polymer; Fabrication Methods, Structure, and Electro-Mechanical Properties,” *RSC Advances* 13 (2023): 370–387, <https://doi.org/10.1039/D2RA06774A>.

33. K.-Z. Tao, B.-W. Zhang, Q. Li, and Q.-F. Yan, “Centimeter-Sized Piezoelectric Single Crystal of Chiral Bismuth-Based Hybrid Halide with Superior Electrostrictive Coefficient,” *Small* 19 (2023): 2207663, <https://doi.org/10.1002/smll.202207663>.

34. L. Pan, H.-F. Ni, P.-Z. Huang, H.-H. Li, M.-J. Lin, G. Teri, J.-Q. Luo, L.-K. Ye, J.-K. Xu, Q.-Q. Jia, C.-F. Wang, Z.-Q. Liu, Z.-X. Zhang, Y. Zhang, and D.-W. Fu, “Multiferroic Orders and Piezoelectric Sensing in an Organic–Inorganic Bismuth Halide,” *Angewandte Chemie International Edition* 65 (2026): e5334832, <https://doi.org/10.1002/anie.5334832>.

35. Y.-Y. He, S.-F. Wu, X.-F. Li, Q. Wang, R.-F. Zhao, L. Pan, C.-B. Qin, X.-M. Zhang, and D.-Y. Fu, “Constructing a Photoferroelectric Semiconductor by Regulating Non-Covalent Interactions through Halogen Substitution,” *Chemical Science* 16 (2025): 20948–20958, <https://doi.org/10.1039/D5SC05946A>.

36. H.-P. Lv, Y.-R. Li, X.-J. Song, N. Zhang, R.-G. Xiong, and H.-Y. Zhang, “A Poling-Free Supramolecular Crown Ether Compound with Large Piezoelectricity,” *Journal of the American Chemical Society* 145 (2023): 3187–3195, <https://doi.org/10.1021/jacs.2c12951>.

37. H.-P. Lv, Z.-L. Liao, D. Gao, K.-W. Jiang, S.-Q. Yu, Y. Lei, J.-C. Liu, Y.-Z. Geng, Y. Ai, P.-F. Li, Y. Qin, and X.-G. Chen, “2D Chiral Organic Salts with a Large Piezoelectric Response,” *Materials Horizons* 13 (2026): 5543–5549, <https://doi.org/10.1039/D6MH00256K>.

38. H. Zhang, Z.-K. Xu, Z.-X. Wang, H. Yu, H.-P. Lv, P.-F. Li, W.-Q. Liao, and R.-G. Xiong, “Large Piezoelectric Response in a Metal-Free Three-Dimensional Perovskite Ferroelectric,” *Journal of the American Chemical Society* 145 (2023): 4892–4899, <https://doi.org/10.1021/jacs.3c00646>.

39. Y.-M. You, W.-Q. Liao, D. Zhao, H.-Y. Ye, Y. Zhang, Q.-H. Zhou, X.-H. Niu, J.-L. Wang, P.-F. Li, D.-W. Fu, Z.-M. Wang, S. Gao, K.-L. Yang, J.-M. Liu, J.-Y. Li, Y.-F. Yan, and R.-G. Xiong, “An Organic–Inorganic Perovskite Ferroelectric with Large Piezoelectric Response,” *Science* 357 (2017): 306–309, <https://doi.org/10.1126/science.aai8535>.

40. K. Robinson, G. V. Gibbs, and P. H. Ribbe, “Quadratic Elongation: A Quantitative Measure of Distortion in Coordination Polyhedra,” *Science* 172 (1971): 567–570, <https://doi.org/10.1126/science.172.3983.567>.

41. M. W. Lufaso and P. M. Woodward, “Jahn–Teller Distortions, Cation Ordering and Octahedral Tilting in Perovskites,” *Acta Crystallographica Section B: Structural Science* 60 (2004): 10–20, <https://doi.org/10.1107/S0108768103026661>.
